# Sputum Microbiota in Tuberculosis as Revealed by 16S rRNA Pyrosequencing

**DOI:** 10.1371/journal.pone.0054574

**Published:** 2013-01-24

**Authors:** Man Kit Cheung, Wai Yip Lam, Wendy Yin Wan Fung, Patrick Tik Wan Law, Chun Hang Au, Wenyan Nong, Kai Man Kam, Hoi Shan Kwan, Stephen Kwok Wing Tsui

**Affiliations:** 1 School of Life Sciences, The Chinese University of Hong Kong, Hong Kong SAR, China; 2 School of Biomedical Sciences, The Chinese University of Hong Kong, Hong Kong SAR, China; 3 Core Facilities Genome Sequencing Laboratory, The Chinese University of Hong Kong, Hong Kong SAR, China; 4 Tuberculosis Reference Laboratory, Department of Health, Hong Kong SAR, China; 5 Hong Kong Bioinformatics Centre, The Chinese University of Hong Kong, Hong Kong SAR, China; Charité-University Medicine Berlin, Germany

## Abstract

**Background:**

Tuberculosis (TB) remains a global threat in the 21st century. Traditional studies of the disease are focused on the single pathogen *Mycobacterium tuberculosis*. Recent studies have revealed associations of some diseases with an imbalance in the microbial community. Characterization of the TB microbiota could allow a better understanding of the disease.

**Methodology/Principal Findings:**

Here, the sputum microbiota in TB infection was examined by using 16S rRNA pyrosequencing. A total of 829,873 high-quality sequencing reads were generated from 22 TB and 14 control sputum samples. *Firmicutes*, *Proteobacteria*, *Bacteroidetes*, *Actinobacteria*, and *Fusobacteria* were the five major bacterial phyla recovered, which together composed over 98% of the microbial community. *Proteobacteria* and *Bacteroidetes* were more represented in the TB samples and *Firmicutes* was more predominant in the controls. Sixteen major bacterial genera were recovered. *Streptococcus*, *Neisseria* and *Prevotella* were the most predominant genera, which were dominated by several operational taxonomic units grouped at a 97% similarity level. *Actinomyces*, *Fusobacterium*, *Leptotrichia*, *Prevotella*, *Streptococcus*, and *Veillonella* were found in all TB samples, possibly representing the core genera in TB sputum microbiota. The less represented genera *Mogibacterium*, *Moryella* and *Oribacterium* were enriched statistically in the TB samples, while a genus belonging to the unclassified *Lactobacillales* was enriched in the controls. The diversity of microbiota was similar in the TB and control samples.

**Conclusions/Significance:**

The composition and diversity of sputum microbiota in TB infection was characterized for the first time by using high-throughput pyrosequencing. It lays the framework for examination of potential roles played by the diverse microbiota in TB pathogenesis and progression, and could ultimately facilitate advances in TB treatment.

## Introduction

Tuberculosis (TB) is an airborne infectious disease caused by the bacterium *Mycobacterium tuberculosis*. In 2010, there were an estimated 8.5 million cases of TB worldwide, causing 1.2 million deaths [1]. Global control of TB has relied on the 90-year-old and largely ineffective Bacille Calmette-Guérin (BCG) vaccine and a few decades-old drugs [2]. The growing epidemics of HIV/AIDS and multidrug-resistant TB strains have further complicated the effectiveness of disease control [3]. As with most classical disease investigations, TB studies have been focused on a single causative agent. However, recent understandings suggest that disease phenotypes are more likely to be a result of complex microbial interactions [4]. Characterizing the whole microbial communities in TB infections might provide further insights into the disease.

The advent of the cloning-independent and massively parallel 454 pyrosequencing technology [5] has facilitated investigations of the soil microbial populations [6], marine microbial communities [7] and the human gut microbiota [8]. Just recently, the microbiota of respiratory diseases such as cystic fibrosis (CF) [9], [10], chronic obstructive pulmonary disease (COPD) [11] and nosocomial pneumonia [12] were also characterized by using this approach. By revealing the composition and diversity of microbiota associated with these diseases, these studies allow the development of hypotheses relating the microbial communities and the disease states. Information on the respiratory microbiota in TB infection is currently unavailable. Here, based on 16S rRNA pyrosequencing, the composition and diversity of sputum microbiota in TB infection was examined for the first time. The microbiota of non-TB case-matched sputum samples was also characterized for comparison.

## Results

A total of 964,556 raw 16S rRNA reads were obtained in this study. About 578,000 reads were from the 22 TB samples and the remaining ∼387,000 reads were from the 14 controls (Table 1). The filtering process removed about 14% of the raw sequencing reads. There were about 499,000 and 331,000 high-quality reads for the TB and control samples, respectively. The average read length was about 370 bp, after removal of the primer sequences. The average number of qualified reads for the TB group was 22,660, and that for the control group was 23,667. Rarifying the number of reads to 13,210, there was an average of 486 and 487 OTUs in the TB and control samples, respectively. The average Chao1 value calculated was 832 in the TB samples and 870 in the controls. The average Shannon index was 4.85 in the TB samples and 4.80 in the controls.

**Table 1:** Patient and sequencing run characteristics.

	TB	Control
Number of patients	22	14
Patient age (median, range)	41, 20–66*	59, 22–82
Patient gender (male/female)	13/8*	6/8
Raw sequencing reads	577,999	386,557
High-quality reads	498,530	331,343
Average reads per sample	22,660	23,667

* Information on age and sex are only available for 21 TB patients.

**Figure 1:**
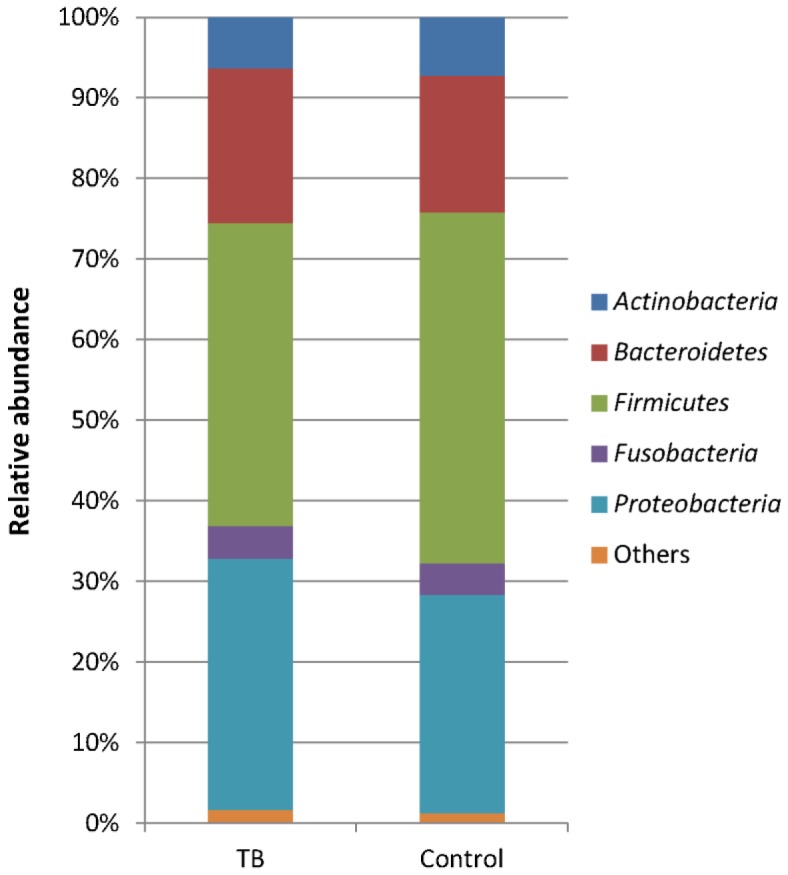
Relative abundance of dominant bacterial phyla in TB and control samples. Only phyla >1% in either group were included.

**Figure 2:**
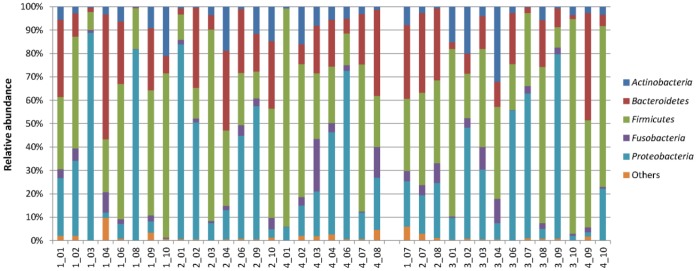
Relative abundance of dominant bacterial phyla in all individuals under study. Only phyla >1% in either group were included. The 22 samples on the left were from the TB patients, and the 14 samples on the right were from the control group.

**Figure 3:**
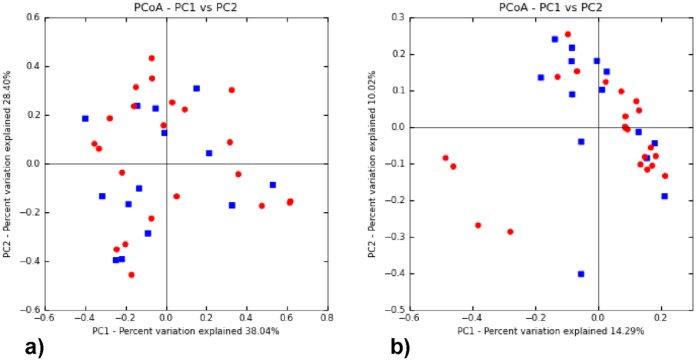
PCoA plots of TB and control samples. The plots were based on a) weighted and b) unweighted UniFrac distances. The two principal coordinates combined explained 66.44% and 24.31% of the variations in the weighted and unweighted cases, respectively. Red dots represent TB samples; blue dots represent control samples.

*Firmicutes*, *Proteobacteria*, *Bacteroidetes*, *Actinobacteria*, and *Fusobacteria* were the major bacterial phyla recovered, which together composed over 98% of the microbial community (Figure 1). *Firmicutes*, *Proteobacteria* and *Bacteroidetes* were among the most abundant phyla in both the TB and control samples, composing 37.6%, 31.2% and 19.2% in the TB samples and 43.6%, 27.1% and 17.0% in the controls, respectively. Whereas the relative abundance of *Actinobacteria* and *Fusobacteria* between the two groups was similar, *Proteobacteria* and *Bacteroidetes* were more represented in the TB samples and *Firmicutes* was more dominant in the controls. Breakdown of the data revealed a large variation in the community structure among the individuals (Figure 2). This was supported by PCoA analysis, in which no obvious differential clustering was observed between members of the TB and control groups (Figure 3).

**Figure 4:**
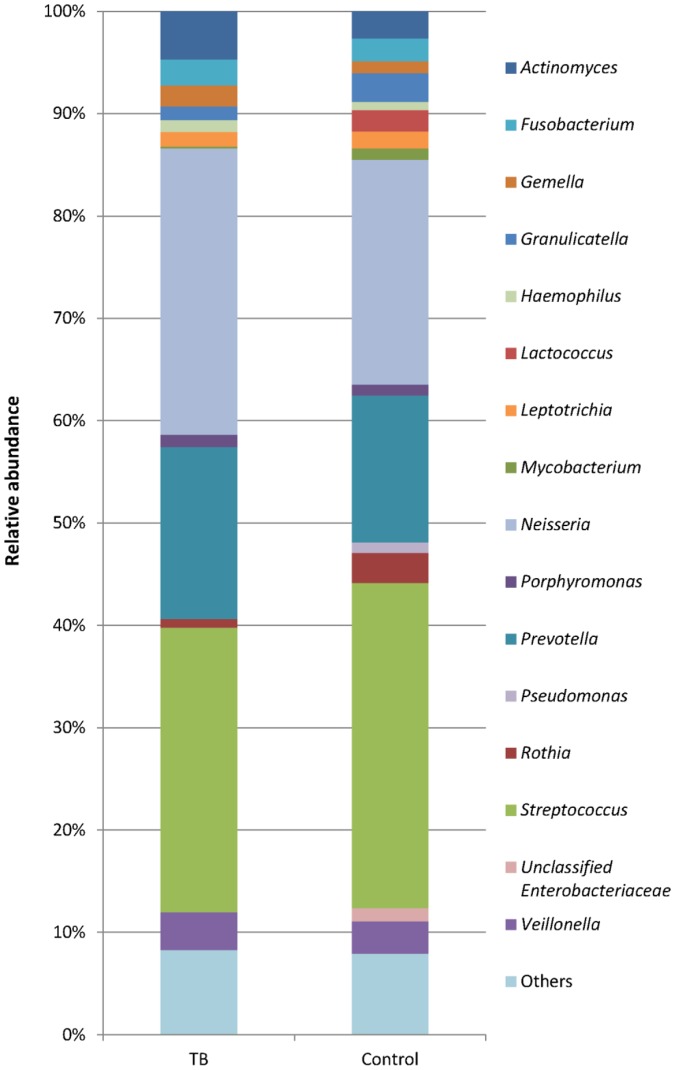
Relative abundance of dominant bacterial genera in TB and control samples. Only genera >1% in either group were included.

**Figure 5:**
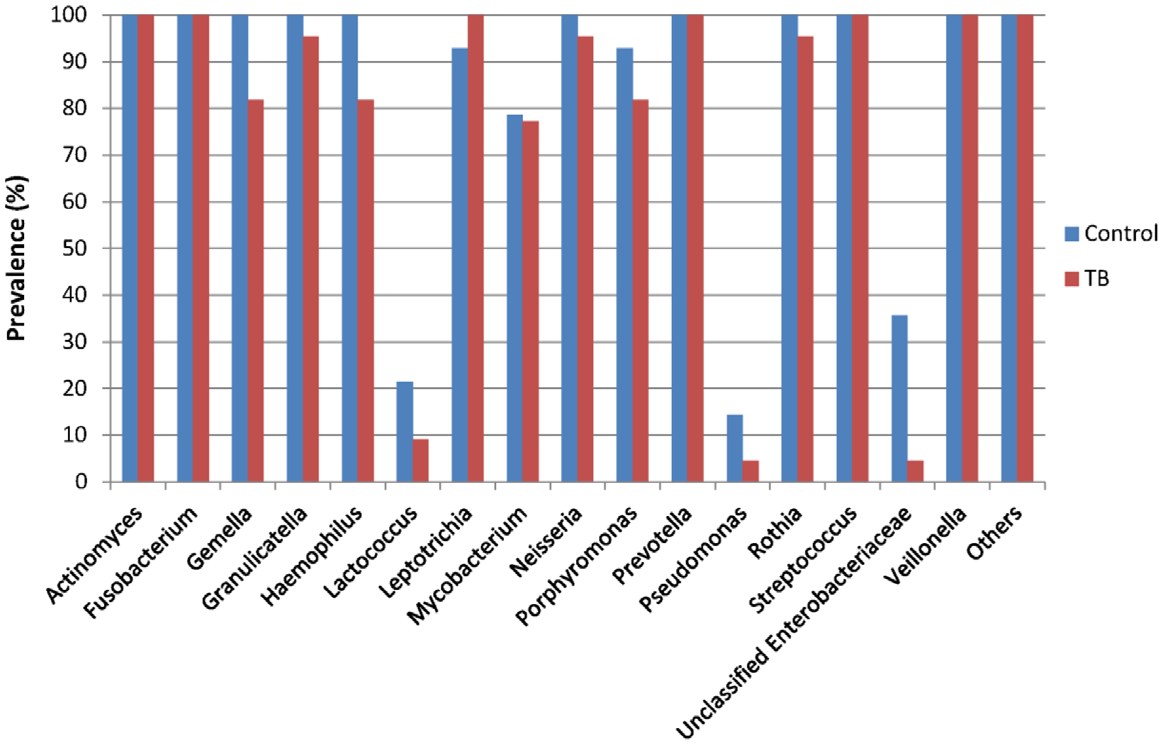
Prevalence of dominant bacterial genera in TB (n = 22) and control (n = 14) samples. Only genera >1% in either group were included.

**Figure 6:**
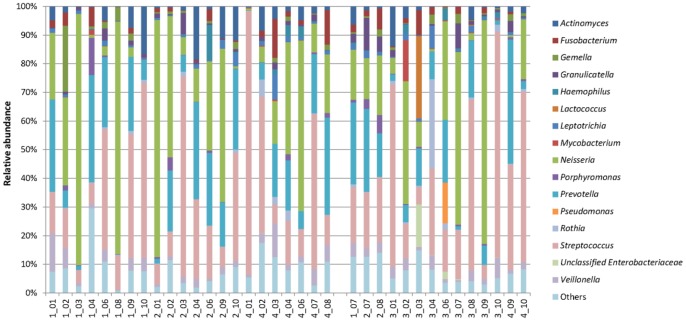
Relative abundance of dominant bacterial genera in all individuals under study. Only genera >1% in either group were included. The 22 samples on the left were from the TB patients, and the 14 samples on the right were from the control group.

Sixteen major bacterial genera were recovered in the sputum samples here (Figure 4). The most abundant genera in the TB samples were *Neisseria* (28.0%), *Streptococcus* (27.8%) and *Prevotella* (16.8%), whereas those for the controls were *Streptococcus* (31.8%), *Neisseria* (22.0%) and *Prevotella* (14.4%). *Neisseria* and *Prevotella* were more represented in the TB samples and *Streptococcus* was more predominant in the controls. *Lactococcus*, *Pseudomonas* and unclassified *Enterobacteriaceae* were less dominant and prevalent in the TB samples (Figures 4 and 5). Within the 16 major genera, only *Actinomyces*, *Fusobacterium*, *Leptotrichia*, *Prevotella*, *Streptococcus*, and *Veillonella* were recovered in all the TB samples, and all these genera besides *Leptotrichia* were found in all the 36 samples (Figure 5). Breakdown of the data again revealed inter-member variations in the community structure at the genus level (Figure 6).

Two OTUs together composed more than 90% of the dominant genus *Neisseria* in both sample groups (Table 2). OTU 6783 was most similar to the uncultured clone NSV3Q1b18 sampled from a showerhead swab [13], and contributed 57.2% and 88.7% of the genus in the TB and control samples, respectively. On the other hand, OTU 7988 was most similar to the uncultured clone 7H59 from the subgingival plaque of a periodontitis-free patient [14], and it was more represented in *Neisseria* in the TB samples (37.4%) than in the controls (4.1%). About one-third of another major genus *Streptococcus* was represented by three OTUs in both sample groups (Table 2). OTU 1734 was most similar to a *S. mitis* clone sampled from the oral cavity [15], and was more represented in *Streptococcus* in the TB samples (12.0%) than in the controls (1.6%). On the other hand, OTU 6370 was most similar to a *S. parasanguinis* clone sampled from the oral cavity, and was more represented in the control samples (16.3%) than in the TB samples (5.6%). OTU 7204 was most similar to the *S. salivarius* strain ATCC 7073, and its contribution in the genus was relatively comparable in both groups (12.4% in TB and 14.6% in control). Two OTUs together composed more than one-third of the genus *Prevotella* in both the TB and control samples (Table 2). They were most similar to uncultured bacterial clones sampled from the human oral cavity.

**Table 2:** List of abundant OTUs composing the three major bacterial genera.

Phylum	Genus	OTU^#^	TB (%)	Control (%)	BLAST result (Identity; isolation source; GenBank accession no.)*
*Bacteroidetes*	*Prevotella*	2225	19.7	26.6	Uncultured clone 071030_015; oral cavity; JQ471460
*Bacteroidetes*	*Prevotella*	7999	14.4	10.7	Uncultured clone 071070_272; oral cavity; JQ476683
*Firmicutes*	*Streptococcus*	1734	12.0	1.6	*Streptococcus mitis* clone; oral cavity; GU422750
*Firmicutes*	*Streptococcus*	6370	5.6	16.3	*Streptococcus parasanguinis* clone; oral cavity; GU412056
*Firmicutes*	*Streptococcus*	7204	12.4	14.6	*Streptococcus salivarius* strain ATCC 7073; NA; NR_042776
*Proteobacteria*	*Neisseria*	6783	57.2	88.7	Uncultured clone NSV3Q1b18; showerhead swab; EU629781
*Proteobacteria*	*Neisseria*	7988	37.4	4.1	Uncultured clone 7H59; subgingival plaque; JX010905

* All with 100% sequence similarity.

In terms of the less represented taxa, genera *Moryella*, *Mogibacterium* and *Oribacterium* were found statistically enriched in the TB samples (*p*<0.05) whereas a genus belonging to the unclassified *Lactobacillales* was enriched in the control samples (*p*<0.05) (Table 3). At the species level, eight OTUs, including those showing 99% identity to *Prevotella melaninogenica*, *Lactobacillus crispatus*, *Streptococcus anginosus*, and *Parvimonas micra*, were found enriched in the TB samples (*p*<0.05). Two other OTUs, including one that is 99% identical to *Aggregatibacter aphrophilus*, were enriched in the control samples (*p*<0.05) (Table 3).

**Table 3:** List of genera and species significantly different between TB and control groups.

Taxonomic level	Identity#			Relative abundance (%)			Enriched in	
	Phylum	Genus	Species (% identity)*	TB	Control	p-value	TB	Control
Genus	*Firmicutes*	*Moryella*	NA	0.1628	0.0420	0.0384	v	
	*Firmicutes*	*Mogibacterium*	NA	0.2187	0.0842	0.0425	v	
	*Firmicutes*	*Oribacterium*	NA	0.3133	0.1460	0.0470	v	
	*Firmicutes*	Unclassified *Lactobacillales*	NA	0.0030	0.0113	0.0286		v
Species∧	*Bacteroidetes*	*Prevotella*	*Prevotella melaninogenica* (99%)	0.0050	0.0003	0.0292	v	
	*Bacteroidetes*	*Prevotella*	*Prevotella histicola* (97%)	0.0056	0.0004	0.0481	v	
	*Proteobacteria*	*Neisseria*	*Neisseria flavescens* (92%)	0.0022	0	0.0413	v	
	*Firmicutes*	*Streptococcus*	*Streptococcus parasanguinis* (92%)	0.0029	0	0.0342	v	
	*Firmicutes*	*Lactobacillus*	*Lactobacillus crispatus* (99%)	0.0227	0.0004	0.0363	v	
	*Firmicutes*	*Streptococcus*	*Streptococcus anginosus* (99%)	0.0063	0.0003	0.0380	v	
	*Firmicutes*	*Streptococcus*	*Streptococcus cristatus* (97%)	0.0138	0.0012	0.0430	v	
	*Firmicutes*	*Parvimonas*	*Parvimonas micra* (99%)	0.1277	0.0118	0.0492	v	
	*Firmicutes*	*Granulicatella*	*Granulicatella adiacens* (92%)	0	0.0011	0.0435		v
	*Proteobacteria*	*Aggregatibacter*	*Aggregatibacter aphrophilus* (99%)	0.0004	0.0045	0.0228		v

∧ OTUs clustered at 97% similarity.

# Using RDP classifier, down to the genus level.

* Against the NCBI 16S rRNA database.

## Discussion

TB remains a global threat in the 21st century. Lead by the World Health Organization, the Stop TB Partnership has planned to halve the global TB prevalence and mortality by 2015 and to eliminate the disease as a public health problem by 2050 [16]. A better understanding of the disease from an angle different from the traditional single-pathogen-based approach could help to meet these targets. Indeed, instead of the presence of a single causative pathogen, it was shown that some diseases are associated with and might result from an imbalance in the microbial community, a condition known as dysbiosis. Inflammatory bowel disease [17] and obesity [18] are two of the well-known examples. The advent of next-generation sequencing technologies, such as 454 pyrosequencing, has greatly advanced studies of the human microbiota [19]. However, unlike the widely studied gut microbiota, the investigation of the lung-associated microbiota is relatively new [20]. In this study, the sputum microbiota in TB infection was characterized by using 16S rRNA pyrosequencing for the first time, aiming to advance understanding of the disease in the microbial community level.

About one million raw sequencing reads were generated for a total of 36 sputum samples in this study. However, the throughput is still insufficient to capture every member in the microbial community. An example particularly worth mentioning is *M. tuberculosis*. Although being the causative agent of the disease, *M. tuberculosis* represents only a very small portion of the whole microbiota in the TB sputum samples (Figure 4). To obtain positive results in the culture and the smear tests, there should be at least 100 *M. tuberculosis* cells/ml and ∼10^4^ acid-fast bacilli (AFB) cells/ml, respectively [21]. Because the average total bacterial load in sputum samples could reach 10^9^ cells/ml [22], 10^5^ reads may be needed to recover a single read of *M. tuberculosis* in a particular TB sample. Since the average sequencing throughput was <27,000 reads per sample here, it is not unexpected that no reads of *M. tuberculosis* were recovered in some of the TB sputum samples. Nevertheless, the sequencing throughput is sufficient to reveal the major phyla and genera of the sputum microbiota, and it allows meaningful comparisons between the TB and control groups.

Five major bacterial phyla were recovered in the sputum samples collected in this study; they were the *Firmicutes*, *Proteobacteria*, *Bacteroidetes*, *Actinobacteria*, and *Fusobacteria*. They are also the predominant phyla in other human body sites, including the oral cavity, skin and colon [19]. In addition, they dominate the microbiota in the CF sputum [10] and the bronchial tract of COPD patients [11]. The three most dominant phyla retrieved in this study (*Firmicutes*, *Proteobacteria* and *Bacteroidetes*) are also the most commonly identified phyla in the normal lung microbiota [20]. These collectively suggest the prevalence of these major phyla in normal and diseased lung microbiota, as well as the human microbiota in general. Within the 16 major genera recovered in this study, only *Actinomyces*, *Fusobacterium*, *Leptotrichia*, *Prevotella*, *Streptococcus*, and *Veillonella* were found in all the TB samples, and they may represent the core genera in the TB sputum microbiota. Similarly, except *Leptotrichia*, the remaining five genera were found in all 36 samples, and they may represent the core genera in the sputum microbiota in general. However, additional studies are needed to confirm this.

*Streptococcus*, *Neisseria* and *Prevotella* were the three most predominant genera recovered in the sputum samples collected in the current study. They are also the major genera retrieved in the sputum of patients with COPD [11], nosocomial pneumonia [12] and CF [9], [10]. This is in contrast to the normal lung microbiota, in which the genus *Pseudomonas* dominates [20]. The genus *Streptococcus* encompasses both commensal and pathogenic Gram-positive bacteria inhabiting various human body sites, including the oral cavity and the upper respiratory tract. *Streptococcus pneumoniae*, for instance, is a well-known pathogen associated with pneumonia [23]. *Neisseria* is a group of Gram-negative bacteria that colonizes human mucosal epithelia. Commensal species include *N. lactamica* and *N. mucosa*, while pathogenic species such as *N. meningitidis* could cause pneumonia [24]. *Prevotella* contains obligate anaerobes commonly isolated from the human oral and intestinal tracts, and it can cause lower respiratory tract infections. Members within this genus are believed to cause pulmonary exacerbations through polymicrobial interactions that enhance the virulence of the established pathogens [25].

Each of the three most predominant genera was dominated by two to three major OTUs that together composed one-third to 90% of the groups. OTU 1734 of the genus *Streptococcus* is 100% identical to *S. mitis* and was more represented in *Streptococcus* in the TB samples than in the controls. Although usually commensal, *S. mitis* could become a significant pathogen in immunocompromised patients as well as individuals implicated in a wide range of diseases, including pneumonia [26]. Indeed, genome study of the *S. mitis* B6 strain has revealed homologues of many previously identified *S. pneumoniae* virulence factors such as autolysins [27]. Also, incubation of *S. mitis* in the gingival epithelial cells was reported to induce the expression of human β-defensin 2 that can kill other oral pathogens [28]. It is thus reasonable to speculate that the increased relative abundance of the opportunistic pathogen in TB infections could alter the microbial community in TB lung and affect TB pathogenesis and progression. Unlike OTU 1734, OTU 6370, which is 100% identical to *S. parasanguinis*, was more represented in the control samples. *S. parasanguinis* is a member of the viridans streptococci that constitute the major population of the human oral microbiota. It is also one of the early colonizers of the tooth surface [29]. OTU 7204 is most similar to *S. salivarius* and was of relatively comparable abundance in *Streptococcus* in both groups. Although being another member of the viridans streptococci, *S. salivarius* commonly causes bacteremia [30]. The two dominant *Neisseria* OTUs and the two dominant *Prevotella* OTUs are most similar to uncultured clones with limited information. Additional research works on these organisms are urged to better understand the potential effects of these abundant members in TB or sputum microbiota.

The less abundant taxa may also affect the dynamics of the microbial community and clinical outcomes [31]. *Mogibacterium*, *Moryella* and *Oribacterium* were the genera enriched statistically in the TB samples collected in this study. *Mogibacterium* is a group of Gram-positive anaerobic bacteria, in which members such as *M. timidum* have been identified in cases of acute lung infections [32]. On the other hand, *Moryella* and *Oribacterium* are genera of anaerobic bacteria that were identified in the recent decade [33], [34]. Little information is available since their discovery. At the species level, eight less represented OTUs were found significantly enriched in the TB samples, each with an over 10-fold difference in relative abundance between the sample groups. Four of them are with high similarity (99%) to known bacterial species, including *Prevotella melaninogenica*, *Lactobacillus crispatus*, *Streptococcus anginosus*, and *Parvimonas micro*. *P. melaninogenica* is a Gram-negative anaerobic bacterium that represents a ubiquitous member of the oral commensal microbiota. However, putative virulence factors such as hemagglutination were reported in some strains [35]. Lactobacilli are Gram-positive bacteria that are usually regarded as beneficial to human health. However, *L. crispatus* strain M206119 was found to exacerbate dextran sulfate sodium (DSS)-induced colitis in mice by interfering with their murine inflammatory responses [36]. It was reported that *S. anginosus* infections might simulate tuberculosis [37]. Indeed, three cases of co-infection by *S. anginosus* and *M. tuberculosis* were reported previously [38]. *P. micra* is a Gram-positive anaerobe usually isolated from the human oral cavity. However, association of the bacterium with respiratory and gastrointestinal tract infections was reported previously [39]. The enrichment of these opportunistic pathogens in the TB sputum revealed here suggests their association with TB pathogenesis or progression.

Bacterial community diversity in CF sputum was found to be positively correlated with the patient stability [9], [36]. It was also reported that a decrease in microbial diversity in the gastrointestinal tract is associated with an increased incidence of inflammatory bowel disease [40]. However, bacterial diversity was found to be significantly higher among asthmatic patients than in controls [41]. In our study, the microbial diversity was found to be similar in the TB and the control sputum samples. Thus, there does not seem to be a general relationship between microbial diversity and the disease state. Indeed, this is not unexpected because different diseases may involve different sets of microbiota, within which interactions could be complex, including both synergistic and antagonistic effects among members [42].

The current study represents the first characterization of the sputum microbiota in TB infection based on 16S rRNA pyrosequencing. The work allows future examination of potential roles played by the diverse microbiota in TB pathogenesis and progression, and it could ultimately facilitate advances in TB treatment. However, additional studies on the uncultured taxa are urged. Further investigations based on metatranscriptomics, metaproteomics and metabolomics on the RNA, protein and metabolite levels, respectively, are also expected to provide further insights into understanding the disease.

## Materials and Methods

### Ethics Statement

The study was approved by the Joint CUHK-NTEC Clinical Research Ethical Committee at the Prince of Wales Hospital in Hong Kong. Written informed consent was obtained from all the studied subjects for sample collection and subsequent analysis.

### Sample Collection and DNA Extraction

Sputum samples were collected from the Tuberculosis Reference Laboratory of the Hong Kong government in a routine TB screening exercise. All samples in this study were from patients who are ethnically Hong Kong Chinese. All the patients were free of HIV. No anti-TB or other antibiotic medication was given to the patients within four weeks of sputum collection. Smear-positive and culture-positive TB sputum samples were collected from 22 patients (13 males and eight females, aged between 20 and 66; data missing for one patient) (Table 1, Dataset S1). Control sputum samples were also collected from 14 individuals (six males and eight females, aged between 22 and 82) with TB-resembling coughing symptoms but being culture-negative for comparison. The sputum samples were treated with a 3% NaOH solution, neutralized with a phosphate buffer and then centrifuged. About 200 µl supernatant was subjected to genomic DNA extraction using the QIAamp DNA Mini Kit (*Qiagen*, Valencia, CA, USA).

### PCR and Pyrosequencing

PCR was performed using composite primers flanking the hypervariable V1–V2 region of the 16S rRNA gene: A-8F (5′-AGAGTTTGATCCTGGCTCAG-3′) and B-357R (5′-CTGCTGCCTYCCGTA-3′) [43]. Sequencing adaptors and sample-specific multiplex identifiers (MIDs) were also added to the 5′ ends of the primers according to the manufacturer’s protocol. PCR reactions were carried out using the Platinum® PCR SuperMix High Fidelity Kit (*Invitrogen*, Carlsbad, *CA, USA*). The PCR thermal regime consisted of an initial denaturation of 30 s at 94°C, followed by 28 cycles of 30 s at 94°C, 30 s at 55°C and 40 s at 68°C. PCR products were purified using the MinElute PCR Purification Kit (*Qiagen*, Valencia, CA, USA) and the quality of the purified products was assessed using Agilent Bioanalyzer 2100. Pyrosequencing of the purified PCR products was performed uni-directionally from the A-8F primer end and on a GS FLX-Titanium platform in a single full-plate run. The sequencing data were submitted to the NCBI short read archive (accession number SRA058505).

### Data Analyses

Raw sequencing reads were demultiplexed, quality-filtered and analyzed using QIIME 1.4.0 [44]. Briefly, reads shorter than 200 bp or longer than 1000 bp, with an average quality score lower than 25 in a sliding window of 50 bp, with mismatching primer sequences, or with ambiguous bases (Ns) were removed from downstream analyses. Quality-filtered reads were clustered into operational taxonomic units (OTUs) at 97% similarity. Taxonomic assignment of representative OTUs was performed using the RDP Classifier [45] at a 0.8 confidence threshold against the Greengenes core set [46]. The dataset was rarified before alpha diversity calculations. Principal coordinate analysis (PCoA) was performed using the weighted and unweighted UniFrac distances [47]. Statistical analyses were performed using SAS.

## Supporting Information

Dataset S1 **Information of patients and samples examined.**
(XLS)
